# The Principle of Least Effort and Comprehension of Spoken Sentences by Younger and Older Adults

**DOI:** 10.3389/fpsyg.2021.629464

**Published:** 2021-03-16

**Authors:** Nicolai D. Ayasse, Alana J. Hodson, Arthur Wingfield

**Affiliations:** Volen National Center for Complex Systems, Brandeis University, Waltham, MA, United States

**Keywords:** sentence comprehension, cognitive effort, pupillometry, syntactic complexity, adult aging

## Abstract

There is considerable evidence that listeners’ understanding of a spoken sentence need not always follow from a full analysis of the words and syntax of the utterance. Rather, listeners may instead conduct a superficial analysis, sampling some words and using presumed plausibility to arrive at an understanding of the sentence meaning. Because this latter strategy occurs more often for sentences with complex syntax that place a heavier processing burden on the listener than sentences with simpler syntax, shallow processing may represent a resource conserving strategy reflected in reduced processing effort. This factor may be even more important for older adults who as a group are known to have more limited working memory resources. In the present experiment, 40 older adults (*M*_age_ = 75.5 years) and 20 younger adults (*M*_age_ = 20.7) were tested for comprehension of plausible and implausible sentences with a simpler subject-relative embedded clause structure or a more complex object-relative embedded clause structure. Dilation of the pupil of the eye was recorded as an index of processing effort. Results confirmed greater comprehension accuracy for plausible than implausible sentences, and for sentences with simpler than more complex syntax, with both effects amplified for the older adults. Analysis of peak pupil dilations for implausible sentences revealed a complex three-way interaction between age, syntactic complexity, and plausibility. Results are discussed in terms of models of sentence comprehension, and pupillometry as an index of intentional task engagement.

## Introduction

Given the ease with which the meaning of a spoken sentence is ordinarily understood, it is easy to overlook the number and complexity of the operations that underlie this success. These operations include extraction and coding of the phonological content from a transient acoustic signal, matching the phonology against potential lexical candidates, and detecting the syntactic structure and semantic relations among these lexical elements to yield an overall understanding of the utterance. It is especially notable that, in spite of the complexity of these operations and age-related declines in speed of processing, executive function, and working memory resources (Hasher et al., 1991; Salthouse, 1994; Salthouse and Meinz, 1995; McCabe et al., 2010), understanding everyday spoken language is among the best preserved of cognitive abilities in adult aging (Wingfield and Stine-Morrow, 2000).

This general success, however, is challenged when older adults are tested for comprehension and recall of sentences that express their meaning with complex syntax that draws heavily on working memory resources (Carpenter et al., 1994; DeCaro et al., 2016). Yet, even as sentence comprehension may show decrements in such cases, there is rarely a catastrophic failure. In this paper, we consider the role of shallow processing as a partial answer to older adults’ relative success in interpreting the meaning of a sentence, and how comprehension strategies may draw differentially on cognitive effort.

The early psycholinguistics literature focused on a platonic ideal in which a full syntactic analysis of the lexical input was conducted, and made possible, access to the sentence meaning (Miller, 1962; Chomsky and Miller, 1963; Fodor and Bever, 1965; Fodor et al., 1974; see also MacDonald et al., 1994). Although comprehension of a sentence may be achieved in this way, it has been argued that under some circumstances correct comprehension of a sentence can be achieved by a route other than a full lexical and syntactic (*lexico-syntactic*) analysis of the stimulus input.

Sometimes called *shallow* processing (Sanford and Sturt, 2002), or processing that is “good enough” for likely comprehension under most circumstances (Ferreira and Patson, 2007; Christianson, 2016), the meaning of a sentence may also be achieved without a full word-by-word lexico-syntactical analysis. Rather, the meaning may be derived by a rapid sampling of some words in the sentence, and using real-world knowledge and presumed plausibility, to guide understanding of the utterance.

That is, rather than the early presumption that there is a single, optimal solution underlying successful comprehension of a spoken sentence, there are multiple solutions to successful sentence comprehension. Prior studies have shown, for example, that when presented with a syntactically complex sentence with an implausible meaning, listeners are more likely to misinterpret the sentence by focusing on plausibility than on its actual meaning, while the opposite is true for such sentences when they have a simple syntactic structure (Ferreira, 2003; Ferreira and Patson, 2007; Amichetti et al., 2016). For example, one would clearly understand the sentence, “The tall man bit the large dog,” however, unlikely this might be. When a sentence has a more complex structure that requires some effort to parse (e.g., “The dog that the tall man bit was large”), a person may assume that the dog bit the man, reflecting the likelihood that the listener took a processing short cut by sampling a few key words and using presumed plausibility (men do not usually bite dogs) to assume its meaning (Erickson and Mattson, 1981; Barton and Sanford, 1993; Ferreira and Patson, 2007).

It is the case that when faced with a syntactically complex sentence such as the above example, the listener may have the ability to process each word as it arrives, determine that the sentence has an embedded clause structure, and resolve the thematic relations among the sentence elements to arrive at a comprehension solution. However, to the extent that these operations come at the cost of cognitive effort, there will be an advantage to an ordinarily adaptive strategy of plausibility based shallow processing.

In this regard, we may, borrowing Zipf’s (1949) term, refer to this alternative strategy as reflecting a *principle of least effort*: that listeners will often, if not invariably, attempt to derive the meaning of an utterance while expending as little effort as possible (for a similar effort conservation argument see, for example, Brehm and Self, 1989; Richter, 2016).

The notion that the listener’s goal is successful comprehension with expenditure of least effort is aligned with general-resource models such as that of Kahneman (1973), who postulated a limited pool of attentional resources that must be allocated among concurrent or closely successive tasks. In such models, effort and resources are intimately related, with effort represented in terms of the resources one is willing or able to allocate to a particular task (Kahneman, 1973; Pichora-Fuller et al., 2016).

Because we live in a plausible world, a comprehension algorithm based on shallow processing with heavy reliance on presumed plausibility will usually lead to a correct interpretation of a sentence meaning. Importantly, this success would ordinarily be indistinguishable from an assumption that a full lexico-syntactic analysis of the sentence had taken place as a precursor to comprehension. Which of the two processing algorithms is being employed, however, can be revealed when comprehension is tested for sentences in which a full analysis of the lexico-syntactic content would yield an implausible meaning (Ferreira, 2003; Padó et al., 2009; Gibson et al., 2013; Amichetti et al., 2016).

As a result of older adults’ more limited working memory resources (Salthouse, 1994; McCabe et al., 2010), one might expect older adults to be more likely than younger adults to use shallow processing. If this is correct, it could potentially be revealed by presenting younger and older adults with syntactically simpler and syntactically more complex sentences that have an implausible meaning. As compared to younger adults, older adults may (a) give fewer correct interpretations as defined by the full lexico-syntactic content, and more misinterpretations of the sentence as having a plausible meaning, and (b) the extent of this age difference should be differentially larger for syntactically complex sentences than for syntactically simpler sentences. Amichetti et al. (2016) have offered evidence in accord with both predictions.

Although the assumption of Amichetti et al. (2016) was that shallow processing of sentences with complex syntax has as its goal comprehension with minimal cognitive effort, the absence of an independent measure of effort left this as a likely but untested hypothesis. The purpose of this present experiment was to apply a direct test of this minimal-effort hypothesis. Although a number of measures have been used to estimate task-related processing effort (McGarrigle et al., 2014), *pupillometry* – the measurement of task-related changes in dilation of the pupil of the eye – offers an objective physiological index of processing effort that does not interfere with conduct of the task itself.

In addition to changes in pupil dilation in response to ambient light and affective stimuli (Kim et al., 2000; Kinner et al., 2017), pupil size has been shown to increase while individuals perform a complex cognitive task (Kahneman and Beatty, 1966; Beatty and Lucero-Wagoner, 2000). Although the mechanisms have yet to be fully understood, it is known that the increase in pupil dilation with cognitive effort or active task engagement is associated with activation in the *locus coeruleus*-norepinephrine (LC-NE) system, thought in turn to modulate prefrontal attentional control (cf., Aston-Jones and Cohen, 2005; Sara, 2009; Reimer et al., 2016).

Specific to our present interests, pupillometry has received wide use as an independent physiological index of processing effort in a variety of language tasks (e.g., Just and Carpenter, 1993; Piquado et al., 2010; Zekveld et al., 2010; see also reviews in van der Wel and Steenbergen, 2018; Zekveld et al., 2018).

In the experiment to be described, healthy community-dwelling older adults heard sentences with a simpler subject-relative embedded clause syntactic structure (e.g., “The eagle that attacked the rabbit was large”), or a more complex object-relative embedded clause syntactic structure (e.g., “The rabbit that the eagle attacked was large”). Counterpart implausible sentences consisting of the same words and syntactic forms were created by switching the agent and recipient of the action (e.g., “The rabbit that attacked the eagle was large” and “The eagle that the rabbit attacked was large”). After a sentence was presented, participants were tested on their understanding of who was the agent or the recipient of the action. Throughout, pupil size was measured, time-locked with what was being heard. A group of younger adults was also included for comparison.

The present experiment had three goals. The first goal was to confirm that, when presented with a sentence in which a full lexico-syntactic analysis would yield an implausible meaning, listeners are more likely to base comprehension on plausibility than on the actual meaning when the sentence has a complex syntactic structure than when it has a simpler structure (e.g., Ferreira and Patson, 2007). The second goal was to confirm that this effect will be greater for older adults than younger adults (Amichetti et al., 2016).

Our primary goal, however, was to test the hypothesis that, when faced with a sentence that has a complex syntactic structure, shallow processing represents an effort-conserving strategy. In this regard we adopt terminology of Pichora-Fuller et al. (2016), in which exertion of effort corresponds to the allocation of processing resources necessary to meet task demands. That is, if shallow processing allows comprehension with less effort, then for syntactically complex sentences with an implausible meaning, one would expect to see a differentially smaller pupillary response when participants give a plausibility based (mis)interpretation of the sentence meaning as compared to when giving a correct interpretation as defined by its full lexico-syntactic content. This might in turn imply that when a sentence has a complex structure, plausibility based shallow processing may yield a likely sentence meaning before a full lexico-syntactic analysis has been completed.

## Materials and Methods

### Participants

The target group consisted of 40 community dwelling older adults (25 females and 15 males), ranging in age from 64 to 89 (*M* = 75.7 years; *SD* = 6.18). For purposes of comparison, we also included a group of 20 younger adults (15 females and 4 males, and one who chose not to disclose). These participants, who were drawn from university students and staff, ranged in age from 18 to 30 (*M* = 20.7 years; *SD* = 2.79).

Audiometric evaluation was carried out for each participant using a Grason-Stadler AudioStar Pro clinical audiometer (Grason-Stadler, Inc., Madison, WI, United States) by way of standard audiometric techniques in a sound-attenuated testing room. The older adults’ mean better-ear pure-tone average (PTA) across 0.5, 1, 2, and 4 kHz was 28.4 dB HL (*SD* = 9.58). Their mean better-ear speech reception threshold (SRT) was 27.6 dB HL (*SD* = 9.67). The younger adults’ mean better-ear PTA was 6.9 dB HL (*SD* = 4.22) and their mean better-ear SRT was 12.3 dB HL (*SD* = 3.43). To accommodate for this common age difference in hearing acuity (Morrell et al., 1996), sound levels were individually adjusted to each individual’s SRT and confirmed for audibility as will be described.

All participants were screened using a 20-item Shipley vocabulary test (Zachary, 1991) to ensure that any potential age differences in task performance would not be due to a chance difference in vocabulary knowledge. The Shipley is a written multiple-choice test in which the participant is asked to indicate which of six listed words means the same or nearly the same as a given target word. It is common for older adults to have superior vocabulary scores compared with younger adults (Kempler and Zelinski, 1994; Verhaeghen, 2003). This held true in the present sample as well [*M* older adults = 15.9, *SD* = 2.41; *M* younger adults = 13.40, *SD* = 1.79, *t*(58) = 4.14, *p* < 0.001].

All participants reported being native speakers of American English with no history of stroke, Parkinson’s disease, or other neurologic involvement that might compromise their ability to perform the research task. Written informed consent was obtained from all participants according to a protocol approved by the Brandeis University Institutional Review Board.

### Stimuli

The sentence stimuli were constructed to reflect two sources of comprehension difficulty based on prior studies: syntactic complexity (e.g., Just and Carpenter, 1992; Cooke et al., 2002; DeCaro et al., 2016) and plausibility (e.g., Obler et al., 1991; Ferreira and Patson, 2007; Amichetti et al., 2016).

Stimulus preparation began with the construction of 128-, 8‐ to 10-word semantically plausible base sentences with a subject-relative embedded clause syntactic structure. Each sentence had an agent of an action, an action, and the recipient of the action (e.g., “The parent that scolded the toddler was tired”). For each of these base sentences, a counterpart sentence was then constructed that contained the same words but that expressed the meaning with an object-relative structure (e.g., “The toddler that the parent scolded was tired”).

To add to the comprehension challenge the length of each of the sentences was increased by insertion of a four-word prepositional phrase into the sentence (e.g., “The parent that scolded the toddler with the striped shirt was tired”). The phrases consisted of a variety of adjectival descriptors and were equally often attached to either the agent or the recipient of the action in the sentence. The phrases were unrelated either to the syntactic form or the plausibility of the sentences, but increased the resultant sentence lengths from the initial 8 to 10 words, to a length of 12 to 14 words.

For each of the subject-relative and object-relative sentences, an implausible version was created by reversing the agent and the recipient the action (e.g., Subject-relative: “The toddler with the striped shirt that scolded the parent was tired”; Object-relative: “The parent that the toddler with the striped shirt scolded was tired”). Example sentences representing the four stimulus conditions are shown in Table 1.

**Table 1:** Example stimuli.

Syntactic structure	Plausibility	Sentence
Subject-relative	Plausible	The parent that scolded the toddler in the striped shirt was tired.
	Implausible	The toddler that scolded the parent in the striped shirt was tired.
Object-relative	Plausible	The toddler in the striped shirt that the parent scolded was tired.
	Implausible	The parent in the striped shirt that the toddler scolded was tired.

A number of accounts have been offered for the heavier processing burden object-relative sentences place on listeners than sentences with a subject-relative structure. These include the non-canonical order of the thematic roles in object-relative sentences (i.e., the first noun is not the agent of the action), thus requiring a more extensive thematic integration than subject-relative sentences (Warren and Gibson, 2002). In addition, because object-relative structures are less common in everyday discourse (Goldman-Eisler, 1968), they violate listeners’ expectations of the order of the thematic roles, thus requiring a potential reanalysis when their non-canonical word order is realized (cf., Levy, 2008; Padó et al., 2009; Gibson et al., 2013). Taken together, this added processing complexity associated with object-relative sentences is known to produce more comprehension errors than subject-relative sentences for young adults (Just and Carpenter, 1992; Cooke et al., 2002), and even to a greater degree for older adults (e.g., Carpenter et al., 1994; Wingfield et al., 2003; Peelle et al., 2010; DeCaro et al., 2016).

In addition to the test sentences, 32 plausibility-neutral filler sentences with subject-relative and object-relative versions were constructed in which reversing the agent and recipient of the action would be equally plausible (e.g., “The runner that noticed the biker with the purple shirt was alert”).

The stimulus sentences were recorded by a female speaker of American English using natural prosody and normal speech rate onto computer sound files using Sound Studio v2.2.4 (Macromedia, Inc., San Francisco, CA, United States) that digitized (16-bit) at a sampling rate of 44.1 kHz. The stimulus sentences and filler sentences were equated for root-mean-square (RMS) amplitude.

### Procedure

Each participant heard 128 test sentences, 32 in each of the four sentence types (plausible and implausible subject-relative and object-relative sentences), plus 32 filler sentences, for a total of 160 sentences. Two seconds after a sentence ended, the participant was presented with a recorded “yes” or “no” comprehension question, such as “Was the parent the do-er of the action?” or “Was the toddler the receiver of the action?” Responses were made by pressing an appropriate key on a keyboard. For a quarter of the trials, participants were also asked a second filler question, such as “Were the do-er and receiver of the action both people?” These filler questions were included to encourage participants to listen to the full sentence before responding (e.g., Ferreira, 2003).

No participant heard any version of a base sentence (a particular combination of agent, action, and recipient) more than once, with each of the versions of each base sentence counterbalanced across participants such that, by the end of the experiment, each of the four versions of each base sentence had been heard an equal number of times. Representatives of the four sentence types and neutral filler sentences were randomly interspersed in presentation. Each trial consisted of 1 s of silence, auditory presentation of a test sentence, then 2 s of silence followed by a recorded comprehension question.

Stimuli were presented binaurally over Eartone insert earphones (E-A-R Auditory Systems, Aero Company, Indianapolis, IN, United States) at 20 dB above each participant’s better-ear SRT. To ensure that the speech materials would be audible to all participants, a pretest was conducted in which 10 words were presented one at a time at the sound level to be used for that participant in the main experiment. The instructions were simply to repeat each word as it was presented. All participants achieved 100% repetition accuracy. The main experiment was preceded by eight practice trials using the same procedures as would be used in the main experiment. None of the sentences used in the practice trials was used in the main experiment.

The large number of experimental sentences was selected to obtain reliable means for the pupillometry data (see Winn et al., 2018). However, breaks were offered to the participant throughout the experiment to avoid fatigue and any possible discomfort from prolonged sitting. The entire study lasted about 2 h.

### Pupillometry Acquisition and Preprocessing

Throughout the course of each trial, the participant’s moment-to-moment pupil size was recorded *via* a desk-mounted EyeLink 1000 Plus eye-tracking apparatus (SR Research, Mississauga, ON, Canada), using a standard 9-point calibration procedure. The EyeLink acquired pupil size data at a rate of 1,000 Hz with data recorded via MATLAB software (MathWorks, Natick, MA, United States). The participants were seated 60 cm from the EyeLink camera, with their head stabilized using a customized, individually adjusted chin rest.

Pupil diameters below 3 SDs of a trial mean were coded as a blink (e.g., Wendt et al., 2016; Winn et al., 2018). Blinks were removed and linear interpolation was performed starting 80 ms before, and ending 160 ms after each blink. This procedure was used to reduce artifacts resulting from partial closures of the eyelids at the beginning and ending of a blink that would cause brief partial obscurations of the pupil (Siegle et al., 2008; Winn et al., 2015). A 20-sample moving average smoothing filter was then passed over the data (Winn et al., 2015, 2018). Pupil size for each trial was baseline-corrected to account for inter-trial pupil size drift by subtracting the mean pupil size over the 1-s pre-sentence silent period from the task-related pupil sizes. Other than blinks no outlier algorithm was employed.

Comparing pupillary responses for individuals who vary in age requires special care because the base pupil size and dynamic range of older adults’ pupils tend to be smaller than that for younger adults (*senile miosis*; Bitsios et al., 1996). Because of this one might underestimate the level of effort allocated to a task by older adults relative to younger adults. To accommodate for this age difference, prior to the main experiment each individual’s pupil size was measured while he or she viewed a light screen (199.8 cd/m^2^) and a dark screen (0.4 cd/m^2^) presented for 60 s each.

Task-related pupil sizes were then represented as a percentage ratio of the individuals’ minimum constriction and maximum dilation as measured in the pre-test (see the discussion in Winn et al., 2018). This was calculated as (*d_M_* − *d_min_*/*d_max_* − *d_min_*) × 100, where *d_M_* was the participant’s measured pupil size at a given time point, *d_min_* was the participant’s minimum constriction, taken as the average pupil size over the last 30 s of viewing the light screen, and *d_max_* was the participant’s maximum dilation measured as the pupil size averaged over the last 30 s of viewing the dark screen (e.g., Ayasse et al., 2017; Ayasse and Wingfield, 2018; Winn et al., 2018). Ambient light in the testing room was kept constant throughout the experiment.

## Results

### Response Accuracy

**Figure 1:**
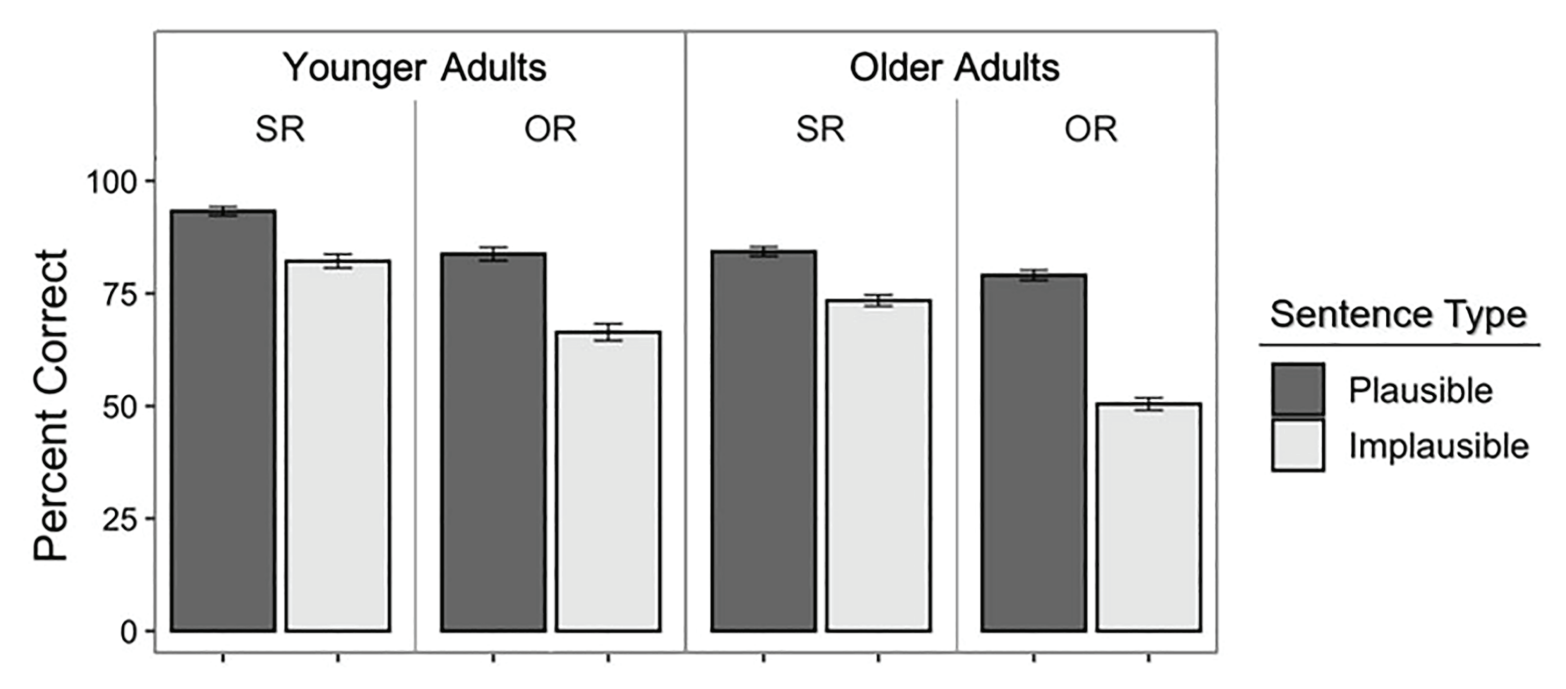
Mean percentage of correct comprehension responses defined as responses in which the judgment of the agent or recipient of an action followed the lexico-syntactic content of the sentence. Data are shown for the younger adults **(left panel)** and older adults **(right panel)** for plausible and implausible subject-relative (SR) and object-relative (OR) sentences. Error bars represent 1 SE.

Figure 1 shows the mean percentage of responses given by younger adults (left panel) and older adults (right panel) that were correct interpretations according to the lexico-syntactic content of the sentence. These data are shown for plausible and implausible sentences when the meaning was expressed with either a subject-relative (SR) or an object-relative (OR) structure.

The data shown in Figure 1 were evaluated using a logistic mixed-effects model as shown in Table 2. Plausibility (plausible and implausible), syntax (subject-relative and object-relative), and age group (younger and older) were included as categorical fixed effects. Participants and items were included as random effects using an intercept-only model based on Matuschek et al. (2017) suggested method for choosing the most parsimonious model. The influences of the fixed effects on model fit were evaluated using model comparisons of the change in log-likelihood using the analysis of variance function (Bates et al., 2015). Plausible was treated as the reference from which the relative parameters for implausible were estimated. For syntax, subject-relative was treated as the reference from which the relative parameters for object-relative were estimated. For age group, younger adults were treated as the reference from which the relative parameters for older adults were estimated. All analyses were carried out in *R* version 3.5.2 using the lme4 package (version 1.1-19) and the function *glmer* to fit the models. The fixed effects and interactions were added into the model in the order shown in the Table 2.

**Table 2:** Predictors of correct interpretations.

Predictor	*B*^a^	*Χ*^2^^b^	df^c^	*p*^d^
Plausibility	−0.57	79.96	1	**<0.001**
Syntax	−0.43	83.70	1	**<0.001**
Plausibility × Syntax	−0.14	12.75	1	**<0.001**
Age Group	−0.38	7.52	1	**0.006**
Plausibility × Age group	−0.00	0.01	1	0.923
Syntax × Age group	0.05	1.97	1	0.161
Plausibility × Syntax × Age group	−0.11	8.95	1	**0.003**

Significant *p* values indicated in bold.

a Unstandardized coefficient of standardized variables.

b *χ*^2^ value for comparisons of each step of the model.

c Degrees of freedom for the *χ*^2^ test.

d Value of *p* reflects significance of change in model fit at each step of the model.

In accord with prior studies (Obler et al., 1991; Ferreira, 2003; Amichetti et al., 2016), the model confirmed a significant main effect of plausibility, reflecting the appearance in Figure 1 of overall greater comprehension accuracy for plausible than for implausible sentences (*p* < 0.001 in all cases). Also in accord with prior studies (Just and Carpenter, 1992; Cooke et al., 2002; Wingfield et al., 2003; DeCaro et al., 2016), there was a significant main effect of syntax, confirming the appearance in Figure 1 of poorer accuracy for object-relative sentences than subject-relative sentences.

The appearance in Figure 1 of the accuracy difference between plausible and implausible sentences being differentially greater for the syntactically more complex object-relative sentences than for the syntactically simpler subject-relative sentences was confirmed by the significant plausibility × syntax interaction shown in Table 2. The older adults’ generally poorer comprehension accuracy as compared to the younger adults was confirmed by a significant main effect of age. Importantly, the above effects were moderated by a significant three-way, plausibility × syntax × age interaction, reflecting the pattern seen in Figure 1 in which the effect of syntactic complexity was larger for implausible sentences and especially so for the older adults as compared to the younger adults. It can be seen in Figure 1 that for the hardest condition (object-relative implausible sentences), the older adults were responding with a lexico-syntactically based correct response vs. a plausibility based misinterpretation at an approximately chance level.

### Pupillary Response and Comprehension Accuracy

As commonly found in pupillometry studies (e.g., Piquado et al., 2010), across all conditions pupil size increased progressively as more and more of a sentence was heard, largely undifferentiated by condition. Pupil sizes associated with conditions, however, dissociated during the 2 s following the end of the sentence, during which time the participant presumably completed the sentence processing and prepared for the comprehension question.

Although our primary interest is the implications for processing effort of listeners’ alternate treatments of implausible sentences in particular, we show in Table 3 mean pupillary responses associated with general comprehension accuracy across age and stimulus conditions. Shown in Table 3 are the mean adjusted peak pupil dilations (PPDs) measured during this 2-s silent period between the end of a stimulus sentence and presentation of the comprehension question for the younger and older adults for correct sentence interpretations based on their lexico-syntactic content, for subject-relative and object-relative plausible and implausible sentences.

**Table 3:** Peak pupil dilations (PPDs) associated with correct interpretations.

Sentence type		Younger adults	Older adults
		Mean (SD)	Mean (SD)
Subject-relative	Plausible	0.073 (0.048)	0.098 (0.083)
	Implausible	0.092 (0.051)	0.114 (0.076)
Object-relative	Plausible	0.085 (0.052)	0.111 (0.080)
	Implausible	0.105 (0.080)	0.115 (0.080)

SD, standard deviation.

The data shown in Table 3 were analyzed with a linear mixed-effects model run in the same manner as for the accuracy data, with the exception of using *lmer* to fit the model. The results of this analysis are shown in Table 4, with the fixed effects and interactions added into the model in the order in which they appear in the table. This analysis confirmed a significant main effect of plausibility, reflecting the overall larger adjusted PPDs when participants gave correct comprehension responses for implausible sentences than plausible sentences. In agreement with previous findings (Just and Carpenter, 1993; Piquado et al., 2010), PPDs were larger with correct responses for object-relative sentences than for subject-relative sentences. Unlike the behavioral accuracy data, however, the analysis of the pupillary responses shown in Table 4 failed yield a significant main effect of age on PPDs, nor were there interactions involving age. In addition, Table 4 fails to show a significant interaction between plausibility and syntax on the pupillary response.

**Table 4:** Predictors of peak pupil dilations for correct interpretations.

Predictor	*B*^a^	*χ*^2^^b^	df^c^	*p*^d^
Plausibility	0.05	11.10	1	**<0.001**
Syntax	0.04	10.56	1	**0.001**
Plausibility × Syntax	−0.00	0.01	1	0.915
Age group	0.07	1.72	1	0.190
Plausibility × Age group	−0.02	1.52	1	0.217
Syntax × Age group	−0.01	0.98	1	0.321
Plausibility × Syntax × Age group	−0.02	2.42	1	0.120

Significant values of *p* indicated in bold.

a Unstandardized coefficient of standardized variables.

b *χ*^2^ value for comparisons of each step of the model.

c Degrees of freedom for the *χ*^2^ test.

d Value of *p* reflects significance of change in model fit at each step of the model.

### Pupillary Responses and Interpretation of Implausible Sentences

**Figure 2:**
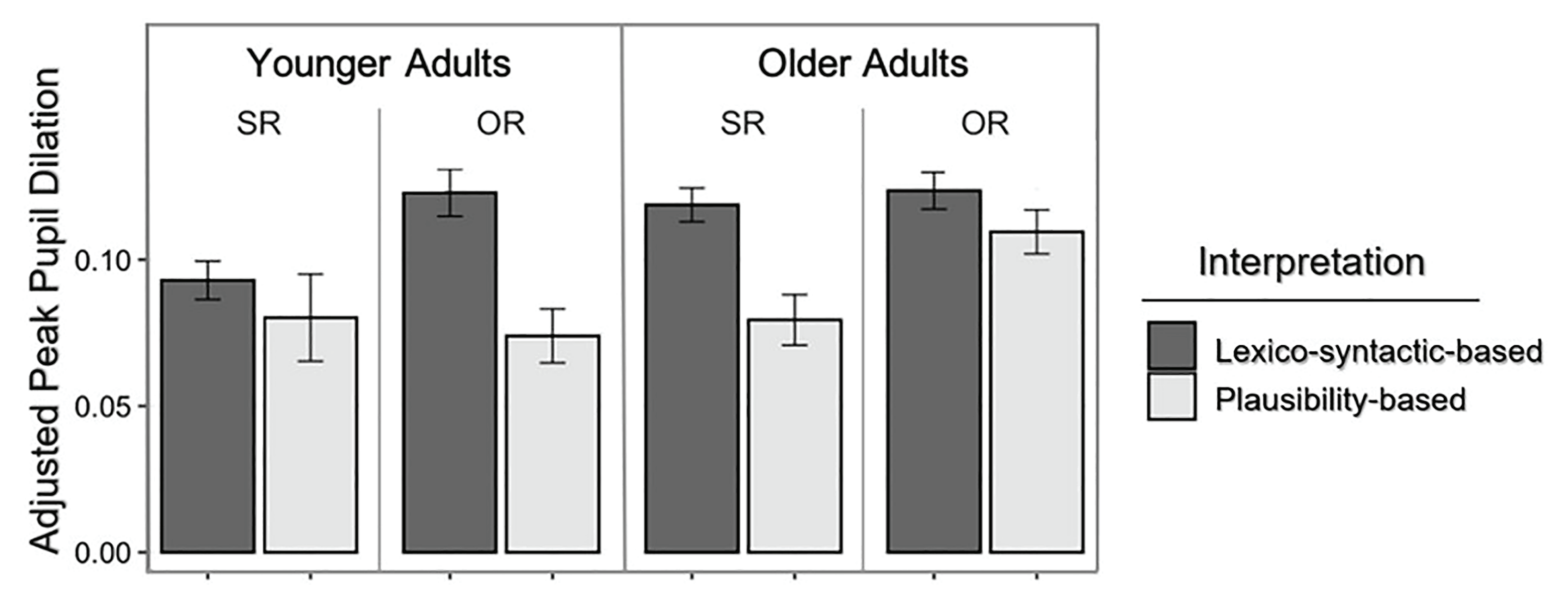
Adjusted PPD associated with implausible SR and OR sentences when younger adults **(left panel)** and older adults **(right panel)** interpreted the sentence meaning consistent with its lexico-syntactic content or gave a plausibility based interpretation. Error bars represent 1 SE.

The apparent paradox of significant influences on behavioral comprehension accuracy that did not appear for PPDs accompanying lexico-syntactically accurate responses was clarified by examining the pupillary responses specifically for the implausible sentences. The implausible sentences hold special interest because listeners may respond either to the actual, albeit implausible meaning as defined by the lexico-syntactic content, or they may misinterpret the sentence as having a plausible meaning. The left and right panels of Figure 2 show mean adjusted PPDs for the younger and older adults for correct interpretations (i.e., interpretations that follow the actual meaning as determined by the lexico-syntactic content of the sentence) and incorrect interpretations (i.e., responses that yielded a plausible interpretation although inconsistent with the actual meaning of the sentence). These data are shown separately for subject-relative (SR) and object-relative (OR) sentences.

The data shown in Figure 2 were analyzed with an additional linear mixed-effects model. The results are shown in Table 5, with the fixed effects and interactions added into the model in the order in which they appear in the table. The results of this analysis fail to show a main effect of age on PPDs. However, age does have an effect, but it is in the form of a three-way interaction in which the effect of age on PPDs is dependent on participants’ particular sentence interpretation, whether lexico-syntactically based or plausibility based, and the sentence syntax, whether the sentence has a subject-relative or an object-relative constriction.

**Table 5:** Predictors of PPDs for interpreting implausible sentences.

Predictor	*B*^a^	*χ*^2^^b^	df^c^	*p*^d^
Interpretation	−0.03	2.80	1	0.094
Syntax	0.04	5.59	1	**0.018**
Interpretation × Syntax	−0.00	0.02	1	0.876
Age group	0.05	1.05	1	0.307
Interpretation × Age	0.01	0.09	1	0.759
Syntax × Age	−0.01	0.56	1	0.454
Interpretation × Syntax × Age	0.04	4.81	1	**0.028**

Significant values of *p* indicated in bold.

a Unstandardized coefficient of standardized variables.

b *χ*^2^ value for comparisons of each step of the model.

c Degrees of freedom for the *χ*^2^ test.

d Value of *p* reflects significance of change in model fit at each step of the model.

The source of this interaction can be seen in Figure 2 in three features of the relationship between age group and PPDs. First, for the younger adults, PPDs for the less demanding subject-relative sentences were similar whichever interpretation the participants gave (*p* = 0.658), while for the syntactically more complex object-relative sentences, an interpretation based on the lexico-syntactic content was accompanied by significantly larger PPDs than when a plausible, albeit incorrect interpretation was given (*p* = 0.011). Second, it can be seen by contrast that the older adults showed this pattern of significantly larger PPDs for lexico-syntactic based responses than plausibility-based responses even for the less complex subject-relative sentences (*p* = 0.028).

The final component underlying the three-way interaction can be seen in the older adults’ PPDs for object-relative sentences, where both lexico-syntactically based and plausibility-based interpretations were accompanied by relatively similar PPDs (*p* = 0.305). That is, to the extent that greater task difficulty is accompanied by a concomitant increase in PPD, one would have expected the PPDs for the lexico-syntactic based interpretations for the older adults to be accompanied by a larger PPD than one observes. It should be recalled, however, that for the object-relative implausible sentences, the older adults’ interpretations were at an essentially chance level. The implications of this finding will be discussed.

## Discussion

### Comprehension Accuracy

There are many everyday instances in which the intended meaning of a message is understood in spite of the meaning actually represented by the wording. A case in point is the frequently cited example, “No head injury is too trivial to be ignored” (Wason and Reich, 1979). Whether seen posted in a hospital emergency room or heard in a medical school lecture, not only does one know the intended meaning (even a small head injury should receive attention), but the poor wording that might imply that all head injuries should be ignored may not even be realized. This is one of many examples of what has been called “pragmatic normalization,” in which the meaning of what we hear is adjusted for compliance with real-world knowledge and plausibility (see the discussion in Sanford and Sturt, 2002).

When sentences are presented in the context of an experimental study, one can expect participants to pay far closer attention to the stimulus than one does in everyday life. Yet, even in the context of a laboratory setting, the behavioral data in the present experiment replicated findings in the extant literature that implausible sentences are more frequently misinterpreted than plausible sentences, with this especially the case for sentences that express their meaning with a complex syntactic structure (e.g., Obler et al., 1991; Ferreira, 2003; Ferreira and Patson, 2007; Amichetti et al., 2016). These results are consistent with the notion that such misinterpretations are the result of plausibility based shallow processing; a search for meaning that, in a plausible world, would not require that every sentence need be fully analyzed word-by-word for its meaning to be determined (cf., Ferreira et al., 2002; Sanford and Sturt, 2002; Levy, 2008; Padó et al., 2009).

A detailed account of the linguistic operations that underly listeners’ difficulty with object-relative structures can be found in, for example, Garraffa and Grillo (2008). Studies involving semantically reversible sentences, such as employed by Garraffa and Grillo, allow a sensitive probe of canonicity effects. As these authors note, however, animacy (or plausibility) effects can be expected to constrain operations. Semantically non-reversible sentences such as those employed in the present experiment represent such a case.

There is a consensus in the aging literature that deficits in the comprehension of sentences with complex syntax in healthy (nonpathological) aging is the result of a processing deficit due to reduced resource availability, rather than to a loss of syntactic knowledge *per se* (Carpenter et al., 1994; Zurif et al., 1995; see also Garraffa and Grillo, 2008). It should be noted that we do not in the present paper address the nature of the resource, or resources, that accounted for the age differences observed. There is, however, a significant body of evidence pointing to working memory limitations as the primary factor underlying age differences in comprehension of written and spoken sentences and discourse (e.g., Norman et al., 1992; Carpenter et al., 1994; Stine-Morrow et al., 2000; DeCaro et al., 2016). In accord with this view, we have suggested that, consequent to older adults’ well-documented working memory limitations (Salthouse, 1994; Park and Payer, 2006; McCabe et al., 2010), when confronted with syntactically complex object-relative sentences, older adults would show a differentially greater tendency than younger adults to base their interpretations on plausibility rather than on the actual sentence meaning as expressed by its full lexico-syntactic content. This behavioral finding (e.g., Amichetti et al., 2016) was replicated in the present study.

Although replicating these behavioral findings affirms their robustness, our major focus in the present study was to test the implied but untested presumption in the study of Amichetti et al. (2016). That is, that arriving at the meaning of a syntactically complex sentence *via* plausibility-based shallow processing will yield a likely meaning that conserves processing effort. Here we used the task-related pupillary response as an objective physiological index of processing effort.

### Pupillary Responses

The analysis of participants’ pupillary responses confirmed two expectations based on the postulate that processing effort will be reflected in an increase in pupil dilation (e.g., Winn et al., 2018; Zekveld et al., 2018). These confirmed expectations appeared in the first of our two analyses of the pupillometry data: the PPDs associated with correct sentence interpretations. This analysis showed larger PPDs associated with correct interpretations of syntactically complex object-relative sentences than syntactically simpler subject-relative sentences. This analysis also revealed larger PPDs when the correct interpretation yielded an implausible meaning than a plausible one. Unlike these effects on behavioral accuracy, however, the effects of syntactic complexity and plausibility on pupil dilation were additive rather than multiplicative.

As was previously noted, the pupillary response did not dissociate among conditions as the speech was arriving, but rather, the dissociation appeared during the 2-s interval following the end of a sentence (see Piquado et al., 2010, for a similar finding). This should not be surprising for spoken sentences being heard at naturally rapid speech rates. Two factors could underlie this finding. The first may be a reflection of estimated delays of between 500 and 1,500 ms for the appearance of a pupillary response after a processing event (Hoeks and Levelt, 1993; Verney et al., 2004). In addition to this physiological lag, it should be borne in mind that speech is a transient signal that moves past the ear literally at the speed of sound. What perceptual and cognitive operations cannot be conducted as the speech is arriving, must be conducted on a fading memory trace of the stimulus after its offset. As such, processing may continue, and reach its culmination, in the brief period after the sentence has been completed.

The appearance of an effect of processing strategy on our index of processing effort during the period after termination of a sentence is consistent with either or both the physiological lag in pupil dilation or the natural lag in the processing operations themselves. Of interest, however, is the extent to which the pattern of the pupillometry responses may inform the question of how plausibility might operate along with syntactic operations in arriving at the meaning of a sentence.

There are two primary classes of sentence processing models, drawn originally from reading studies, which have been proposed to explain how listeners may employ plausibility in sentence processing. “Syntax-first” models postulate that listeners first process sentence meaning based purely on lexico-syntactic information, with any check on plausibility occurring after this analysis has been performed (Rayner et al., 1983; Steinhauer et al., 1999). However, according to the second class of models, referred to as “constraint-based” models, syntactic parsing is conducted along with context and real-world knowledge. That is, lexico-syntactic and plausibility based gist analyses are performed essentially in tandem, with plausibility checks occurring as the lexico-syntactic analysis is being performed (Trueswell et al., 1993; MacDonald et al., 1994; Tanenhaus et al., 1995).

In terms of temporal operations, in the case of computationally complex sentences, such as the object-relative constructions employed here, one may expect gist-based comprehension to occur before slower in-depth syntactic representations become fully developed. Once a plausible interpretation has been reached, syntactic operations with attendant demands on processing resources may thus have no need to continue beyond this point (see the discussion in Karimi and Ferreira, 2016; see also Dwivedi, 2013, for analogous arguments for interpreting sentences with quantifier scope ambiguities). This may reasonably be modulated by external factors, such as whether there may be a steep penalty for a comprehension error.

It could be argued that a parallel analysis of syntax and plausibility, an approach consistent with constraint-based models, would likely demand less effort than a syntax-first model when sentences are complex. This would be so to the extent that this approach would allow a lexico-syntactic analysis to be aborted as soon as a likely plausible meaning appears, thus avoiding a full resource-demanding syntactic analysis.

Our present data cannot easily distinguish between syntax-first and constraint-based accounts of how plausibility enters into sentence comprehension. This is so as both models might lead to the expectation of increased processing effort when the integration of plausibility into the comprehension process reveals an implausibility. This shared expectation would be consistent with the present finding of larger PPDs associated with correct comprehension responses for implausible sentences than plausible ones.

In its simplest form, the principle of least effort assumes a link between effort, indexed here by pupil dilation, and task difficulty. In the present experiment, task difficulty was defined by the syntactic complexity of the sentence and its plausibility. As our data showed, however, the link between processing effort and task difficulty is also affected by the solution path taken by the listener: in this case, whether the listener based his or her understanding of the sentence on a full lexico-syntactic analysis of the sentence input, or on a superficial “shallow” analysis of the input, supported by presumed plausibility of the utterance.

The hypothesis that a plausibility-based analysis represents an effort-conserving strategy as compared to a lexico-syntactic analysis that over-rides plausibility, was explored by focusing specifically on the pupillary responses to implausible sentences. It is, in this condition, that one may contrast pupillometry-indexed processing effort when the listener correctly interprets the sentence based on its lexico-syntactic content, or instead gives a plausibility based response indicative of shallow processing. As we discovered in this analysis, the pattern of the pupillary responses to these conditions was a complex one, carried primarily by a three-way interaction in which the pupillary response, although affected by whether a lexico-syntactic based or plausibility based interpretation was given by the participant, was moderated by the syntactic complexity of the stimulus sentence and participant age.

Unpacking this interaction revealed several major principles that emerge from the participants’ pupillary responses. Although it is well-established that successful comprehension of object-relative sentences imposes greater processing demands than subject-relative sentences (e.g., Just and Carpenter, 1992; Cooke et al., 2002; DeCaro et al., 2016), this must be seen in the light of the equally well-established age difference in available resource capacity (Salthouse, 1994; Salthouse and Meinz, 1995). Using pupil dilation as a measure of processing effort, it was seen that when the younger adults used plausibility to determine their understanding of an object-relative sentence less effort was expended than when the meaning was in accord with the actual meaning as determined by its lexico-syntactic content. This same pattern was observed for the older adults, but for the subject-relative sentences that, for these participants, may represent a high demand given available resources. The impact of resource availability was also seen in the younger adults’ pupillary response to the less syntactically complex subject-relative sentences, which for them, required little more effort whether their response was based on a presumed full lexico-syntactic analysis or a response based primarily on plausibility.

This analysis of the pupillary responses to the implausible sentences, however, revealed an important qualification to the difficulty-effort relationship embodied in a principle of least effort. This qualification appeared in the present experiment in the form of a plateau of pupil size from the second-most difficult condition (implausible subject-relative sentences) to the most difficult condition (implausible object-relative sentences) for the older adults. A plateau, or even a dip, in pupil size when task difficulty begins to exceed processing capacity is not without precedence in the literature (e.g., Peavler, 1974), although it has usually been observed in the context of processing effort attendant to perception of acoustically degraded speech, whether due to noise-masking or hearing loss (e.g., Kuchinsky et al., 2013, 2014; Ohlenforst et al., 2017; Wang et al., 2018). In the present case, the plateau appears for implausible object-relative sentences, where the older adults’ attempted interpretation of these doubly complex sentences was at an approximately chance level.

Such results suggest that effort, as estimated by pupil dilation, will increase until a tipping point of difficulty, or perceived difficulty, is reached (Ayasse and Wingfield, 2018). That is, as persuasively argued by Richter (2016), individuals will engage effort, and hence an allocation of resources (Pichora-Fuller et al., 2016), only to the extent that they believe that additional effort will bring success.

Using the framework of behavioral economics, Eckert et al. (2016) have put forth a similar argument in the context of expending effort when trying to understand speech heard in noise. Like Richter, they assume an interdependence between the commitment of effort and likely payoff, suggesting that effort may not be expended if there is an unlikely return on investment. In the Eckert et al. (2016) domain of interest this return would be measured in perceptual success. The suggestion that intentional task engagement may be the driver of the pupillary response has attracted increasing attention in the literature (e.g., Winn et al., 2018).

### Caveats

We offer the present data and interpretations with two caveats. The first is that our current design did not address degrees of implausibility. Measurements of eye-fixations in reading, for example, have shown that the response to an anomalous (impossible) word in a sentence context is significantly faster than the response to a possible, but unlikely word (Rayner et al., 2004; see also Patson and Warren, 2010; Matsuki et al., 2011; Milburn et al., 2015). It remains an open question whether varying the degree of plausibility in an experiment such as the present one might affect the pupillary response.

The second caveat is that in the present experiment, we intentionally focused on linguistic features that especially challenge older adults’ sentence comprehension. It should be borne in mind, however, that in everyday discourse sentences with relatively simple syntax far outnumber those with complex structures (Goldman-Eisler, 1968). This, and adept use of linguistic context, are additional reasons why comprehension in everyday conversations can show minimal if any age differences (Tun and Wingfield, 1993). In a similar way, issues such as differences in verb attachment in garden path sentences can affect the likelihood of shallow processing and whether one will observe age differences in their comprehension (Christianson et al., 2006). It should also be noted that sensory or cognitive challenge may also affect strategic processing, especially among older adults (Kurthen et al., 2020).

Finally, although our focus in this study was on comprehension at the sentence level, Rönnberg et al. (2013) have suggested that external processing constraints, such as placing an individual under time pressure, may result in the listener settling for gist processing at the level of extended discourse, as well as possibly limiting the completeness of processing of all bottom-up features of the acoustic signal itself.

Consistent with the Rönnberg et al. (2013) emphasis on the context in which processing is conducted, is the finding that the typically larger pupillary response for syntactically non-canonical sentences that appears when listeners know they will be tested for comprehension or recall (e.g., Just and Carpenter, 1993; Piquado et al., 2010; Ayasse and Wingfield, 2018) may not appear when participants are told simply to listen to sentences for their meaning (Chapman and Hallowell, 2020). In this regard, Chapman and Hallowell suggest that the anticipation of comprehension questions may encourage a more complete syntactic analysis of sentence stimuli than potentially shallow “good-enough” processing when no test of comprehension is specifically anticipated.

This allusion to the above cited effects of task demands on completeness of syntactic processing (a real or perceived penalty for a comprehension error, a belief in the likelihood of processing success, and weighing accuracy against the value of rapid processing) may address the finding in this and related studies that participants in a particular experimental condition may on average favor a lexico-syntactically based or plausibility based comprehension solution, but that counter examples nevertheless also occur on some trials under the same conditions (cf., Ferreira, 2003; Ferreira and Patson, 2007; Amichetti et al., 2016; Christianson, 2016; see also Dwivedi, 2013). This suggests that although participants’ goal is comprehension with minimal effort, it is a goal tempered by a desire for accuracy.

Of special interest in this regard is the older adults’ performance on the object-relative implausible sentences. If the older adults’ goal was a comprehension solution with the least demand on resources, one would expect to see object-relative implausible sentences yielding uniformly incorrect response in terms of lexico-syntactic content due to a plausibility based solution, rather than the approximately chance level accuracy as observed here, in which examples of both solutions appeared. It may be that the perceived difficulty of the comprehension task led to essentially random agency assignment. On the other hand, although ideally each trial should be treated as an independent event by a study participant, there is a deep literature from several domains showing that individuals’ perceived performance on one task trial can influence performance and/or shift one’s confidence level on a subsequent trial (cf., Begg et al., 1989; Benjamin et al., 1998; Kornell et al., 2011). This may induce a level of variability (“noise”) in decision strategies across trials. We suggest this as a potential area for future research.

## Conclusion

Wason and Reich (1979) referred to their emblematic sentence, “No head injury is too trivial to be ignored” as a “verbal illusion” because most people derive a meaning that is not actually conveyed by its lexico-syntactic content. It is possible that the anomaly present in Wason and Reich’s exemplar sentence overloads the parser, but instead of causing a breakdown in understanding, the anomaly is resolved by a reversal of meaning (Kizach et al., 2016). This remains a possibility. Along with others, however, we suggest that shallow processing is in fact a processing preference; a first-pass system that, besides making possible rapid comprehension with minimal effort, also allows for pragmatic normalization and an ability to understand a speaker’s (or writer’s) intended meaning (Sanford and Graesser, 2010; Christianson, 2016).

In the present experiment, we have highlighted the interplay between syntactic complexity, plausibility, and cognitive effort in sentence comprehension among older and younger adults. In so doing, it is important to note that the speech materials were presented under ideal listening conditions and in the absence of distraction. As such, we may well have underestimated the relative frequency with which shallow processing and presumed plausibility underlies everyday understanding of spoken discourse.

## Data Availability Statement

The raw data supporting the conclusions of this article will be made available by the authors, without undue reservation.

## Ethics Statement

The study protocol was reviewed and approved by the Brandeis University Institutional Review Board (IRB). The participants provided their written informed consent to participate in this study.

## Author Contributions

NA, AH, and AW collaborated on the experimental design, data analysis, data interpretation, and drafting of this manuscript. All authors contributed to the article and approved the submitted version.

### Conflict of Interest

The authors declare that the research was conducted in the absence of any commercial or financial relationships that could be construed as a potential conflict of interest.
